# Reporting reimbursement price decisions for onco-hematology drugs in Spain

**DOI:** 10.3389/fpubh.2023.1265323

**Published:** 2023-10-24

**Authors:** David Elvira, Ferran Torres, Roser Vives, Gemma Puig, Mercè Obach, Daniel Gay, Daniel Varón, Thais de Pando, Josep Tabernero, Caridad Pontes

**Affiliations:** ^1^Departament de Farmacologia, de Terapèutica i de Toxicologia, Universitat Autònoma de Barcelona, Sabadell, Barcelona, Spain; ^2^Sanofi, Paris, France; ^3^Biostatistics Unit, Medical School, Universitat Autònoma de Barcelona, Cerdanyola del Vallés, Barcelona, Spain; ^4^Gerència del Medicament, Servei Català de la Salut, Barcelona, Spain; ^5^Institut Català d’Oncologia, Barcelona, Spain; ^6^Digitalization for the Sustainability of the Healthcare System (DS3), Servei Català de la Salut, Barcelona, Spain; ^7^Vall d’Hebron Hospital Campus and Institute of Oncology (VHIO), Barcelona, Spain

**Keywords:** health technology assessment, multicriteria assessment methods, price and reimbursement systems, onco-hematologic prices, value assessment

## Abstract

**Introduction:**

Even using well-established technology assessment processes, the basis of the decisions on drug price and reimbursement are sometimes perceived as poorly informed and sometimes may be seen as disconnected from value. The literature remains inconclusive about how Health Technology Assessment Bodies (HTAb) should report the determinants of their decisions. This study evaluates the relationship between oncology and hematology drug list prices and structured value parameters at the time of reimbursement decision in Spain.

**Methods:**

The study includes all new onco-hematological products (22), with a first indication authorized between January 2017 and December 2019 in Spain and pricing decisions published up until October 2022. For each product, 56 contextual and non-contextual indicators reflecting the structured multiple criteria decision analysis (MCDA) – Evidence-based Decision-Making (EVIDEM) framework were measured. The relationship between prices and the MCDA-EVIDEM framework was explored using univariate statistical analyses.

**Results:**

Higher prices were observed when the standard of care included for combinations, if there were references to long-lasting responses, for fixed-duration treatment compared to treatment until progression and treatment with lower frequencies of administration; lower prices were observed for oral administration compared to other routes of administration. Statistically significant associations were observed between prices and the median duration of treatment, the impact on patient autonomy, the ease of use of the drug, and the recommendations of experts.

**Discussion:**

The study suggests that indicators related to the type of standard of care, references to long-lasting responders, the convenience of the use of the drug, and the impact of treatment on patient autonomy, as well as contextual indicators such as the existence of previous clinical consensus, are factors in setting oncology drug prices in Spain. The implementation of MCDA-EVIDEM methodologies may be useful to capture the influence on pricing decisions of additional factors not included in legislation or consolidated assessment frameworks such as the European Network for Health Technology Assessment (EunetHTA) core model. It may be opportune to consider this in the upcoming revision of the Spanish regulation for health technology assessments and pricing and reimbursement procedures.

## 1. Introduction

Concerns about the increasing cost of oncological and hematologic innovation in Europe are growing as prices of cancer drugs are high but not always related to a proportional improvement in patient health status (1). In Europe, the increase in the rate of health spending on cancer has been faster than the increase in cancer incidence during the last 20 years. Similarly, the loss of productivity related to premature cancer mortality has decreased, while productivity loss related to morbidity is still uncertain (2).

Progressively flexible regulatory criteria for authorization in the setting of precision medicine aims to accelerate market access decisions at the pricing and reimbursement process. Studies of authorization decisions in Europe have estimated that after monitoring post-authorization real-world evidence for 3.3 years, benefits on survival of those authorized drugs were only observed in 7% of cases, and improvement in reported quality of life was achieved in only 11% of them (3). A recent study (4) confirms that this trend is consolidated, and regulatory practice is biased toward earlier access at the expense of the production of post-authorization robust evidence, especially when the drug covers clinical unmet needs in diseases with poor prognosis (5). Pricing and reimbursement decisions are tough when evidence is scarce and lacking comparative data, risking opportunity costs (6). In order to minimize this, new access management models have been implemented across Europe during the last decades (7), although to a limited extent and with a lack of methodological harmonization (8). The increase in prices of oncologic products has generated additional international concerns (9) about the disconnection between price and value.

There is still an open debate in Europe about which are the adequate methods to assess the value of drugs (10). Methods of setting “fair prices” are generally focused on clinical benefits or expanded to the so-called value-based pricing, which is usually focused on cost-effectiveness analysis (11–13). Cancer drugs are normally classified as innovation based on implicit clinical value through Quality-Adjusted Life-Years – QALYS (e.g., United Kingdom, Australia, Sweden) or using innovation scales (e.g., Canada, Japan, France, Germany, Austria, Italy) (14). However, healthcare authorities do not normally unveil the details of the methodology applied to assess value, while new cancer drugs are increasingly reimbursed at a higher price than the available alternatives (15).

Recent studies (16) show that even in countries with well-established technology assessment processes (such as the UK, Germany, France, and Switzerland), prices may still be considered disconnected from value. In fact, in countries such as France, Australia, or the UK, prices are only weakly associated with drug clinical benefits (17–19).

Besides a lack of elements to check consistency between price and value, the literature remains inconclusive about the factors that Health Technology Assessment Bodies (HTAb) are using to make their decisions on value and how the payers are deciding and reporting price decisions, especially when applying managed entry agreements (20). Recent studies (21) show that EVIDEM’s framework provides a complete and suitable value assessment framework, including contextual dimensions, and it has been progressively adopted by some HTAb in Europe. Additionally, differences may exist in the concept of value between payers and patients: while payers are generally focused on objective clinical outcomes to determine reimbursement conditions, the importance of patient preferences is not clear (22, 23).

In Spain, the pricing and decision process starts after the European marketing authorization is formally adopted by the Spanish Agency of Medicines and Medical Devices (AEMPS) (24). Subsequently, a Therapeutic Positioning Report (TPR) is issued by the REvalMed network (25) to inform about the added therapeutic value of the drug compared to current therapeutic alternatives. The TPR includes a therapeutic evaluation from the AEMPS, an economic assessment from the General Directorate for Common Portfolio of the NHS and Pharmacy Services (DGCCSF), and a final technical revision by external experts and scientific societies appointed by the REvalMed network. The TPR, together with the application dossier filed by the marketing authorization holder and DGCCSF’s reports, is supposed to be the main driver for reimbursement decisions. The Inter-ministerial Committee on Pricing of Medicines and Healthcare Products (CIPM) is the body responsible for the final resolution of price and reimbursement conditions (26). The CIPM decision is published as a listed price (not net price) and motivation in general terms, which are based on the criteria listed in the RDL 1/2015, but the information provided by the Ministry of Health (MoH) is not detailed enough to know how the value of the drug has been established. It has been questioned whether the Spanish pricing model is based only on budgetary impact and lower European nominal price, without accounting for contextual criteria and societal needs.

Detailed information on how Spanish healthcare authorities define price and reimbursement conditions of new drugs is not available, and a lack of predictability, potentially leading to inconsistency between value and price, has been alleged (27, 28). The Royal Legislative Decree 1/2015 (RDL 1/2015) of the Law on Guarantees and Rational Use of Medicines and Health Products (29) lists only a restricted set of criteria to be used by the Spanish National Health System to establish prices of publicly funded medicines.

Based on recent data released by MoH (30), 90% of assessed oncologic medicines in Spain are publicly funded, with a listed price 15 times higher than the average price of new non-cancer-related drugs. By 2021, cancer drug costs represented 16.9% of the global pharmaceutical Spanish public budget, and the cost of cancer drugs at the hospital level has grown by 105.9% since 2016. The main objective of this study is to externally evaluate whether there is a relationship between the prices of oncology and hematology drugs and the evidentiary and contextual information available at the time of reimbursement decision in Spain by applying a structured assessment of parameters measuring drug value and to identify the most relevant criteria related to price decisions made by health authorities.

## 2. Materials and methods

All new chemical entities with a first EMA authorization for a single onco-hematologic indication between January 2017 and December 2019 were identified, and price and reimbursement decisions of the Spanish MoH, including the notified price and public funding authorization, were tracked based on the publicly available database Bifimed (31) and the resolutions published by the MoH up until the end of October 2022 (Supplementary Table 2).

For standardization and comparison purposes, a daily treatment cost based on notified prices was assigned following the Summary of Product Characteristics recommended posology for the studied indication. When the treatment duration was fixed, the cost was annualized. Products with a negative decision were assigned a price of zero; no other data imputation was applied.

For each product, a set of indicators from the MCDA-EVIDEM framework was used. A literature review was carried out to identify the indicators (32–36) for each MCDA-EVIDEM dimension (Table 1). The inclusion criteria for the review were articles published from January 2017 to December 2021 that included MCDA-EVIDEM-related indicators to assess onco-hematologic drugs as well as country legislation and HTAb official documents available in English or Spanish. The review did not include outdated documents. The indicators for each product were extracted from available European Public Assessment Report (EPAR), TPR (37), European Society of Medical Oncology-Magnitude of Clinical Benefit Scale (ESMO-MCBS) evaluations (38), National Institute for Health and Care Excellence (NICE) economic assessments (39), and freely available information from national and regional healthcare authorities (40). The indicators were informed by a stepwise approach including two independent reviewers for each product, and discrepancies were resolved through discussion. Public notified reimbursed prices per product (expressed as annual cost per treatment) were also included.

**Table 1:** Description of MCDA-EVIDEM dimensions and metrics.

Dimensions and indicators	Metrics	Mean (SD) or %	(N)
Noncontextual			
Disease severity			
*Speed tumor growth*	*Time of duplication (months)*	*13.64 (19.61)*	*20*
*% Metastasized*	*Percentage of patients with metastasis at diagnosis*	*50% (40%)*	*22*
*Expected survival 5-years*	*Percentage of patients with expected survival ≥ 5 years*	*29% (25%)*	*22*
*Physical function and general health*	*Normalized Score of SF36 – EQ5D – EORTC QLC or C30*	*62.41 (21.72)*	*12*
Size of affected population			
*Prevalence*	*Cases per 10,000 inhabitants*	*23.83 (219.32)*	*22*
*Incidence*	*New cases per 10,000 inhabitants and year*	*27.06 (29.57)*	*22*
Unmet needs			
*Treatment options*	*Percentage with/without alternative treatment options*	*With: 90%* *Without: 9%*	*22*
*Type of standard of care*	*Percentage of chemotherapy/immunotherapy/directed agents/surgery/radio/combined/others/none*	*Chemotherapy:21%* *Directed agents: 47%* *Combined:17%* *Others: 4%* *None: 9%*	*22*
Comparative effectiveness			
*Progression-free survival*	*Months (median) during which patients have not experienced disease progression*	*13.69 (7.83)*	*22*
*Progression-free survival* vs. *control*	*Difference in months (median) during which patients have not experienced disease progression* vs. *control*	*6.73 (4.59)*	*22*
*Objective response rate (RECIST/MRD)*	*Percentage of patients that experience complete response and partial response*	*0.55 (0.17)*	*19*
*Objective response rate (RECIST/MRD)* vs. *Control*	*Difference in percentage of patients that experience complete response and partial response* vs. *control*	*20% (14%)*	*14*
*Complete response (RECIST/MRD)*	*Percentage of patients that experience complete response*	*23% (27%)*	*20*
*Complete response (RECIST/MRD)* vs. *control*	*Difference in percentage of patients that experience complete response* vs. *control*	*9% (13%)*	*15*
*Partial response (RECIST /MRD)*	*Percentage of patients that experience partial response*	*33% (18%)*	*18*
*Partial response (RECIST /MRD)* vs. *control*	*Difference in percentage of patients that experience partial response* vs. *control*	*10% (7%)*	*13*
*Long responders*	*Percentage of patients mentioned as long responders*	*Yes: 9%* *No: 91%*	*22*
*Overall survival*	*Months (median) of treatment randomized to death*	*25.61 (16.43)*	*15*
*Overall survival* vs. *control*	*Difference in months (median) of treatment randomized to death* vs. *control*	*9.23 (13.25)*	*12*
Comparative safety and tolerability			
*Any adverse event*	*Percentage of patients experiencing an adverse event*	*97% (6%)*	*22*
*Any adverse event* vs. *control*	*difference in percentage of patients experiencing an adverse event* vs. *control*	*5% (10%)*	*16*
*Non-fatal serious adverse events (>3)*	*Percentage of patients experiencing an adverse event of grade 3 to 5*	*57% (26%)*	*16*
*Non-fatal serious adverse events (>3)* vs. *control*	*Difference in percentage of patients experiencing an adverse event of grade 3 to 5* vs. *control*	*15% (19%)*	*16*
*Fatal adverse events (Grade 5)*	*Percentage of patients experiencing an adverse event of grade 5*	*7% (7%)*	*21*
*Fatal adverse events (Grade 5)*	*Difference in percentage of patients experiencing an adverse event of grade 5* vs. *control*	*1% (5%)*	*16*
*Dosage adjustment due to adverse events*	*Mention (yes/no) of dosage adjustment due to adverse effects*	*Yes: 73%* *No: 14%* *Not relevant: 13%*	*22*
*Treatment discontinuation due to adverse events*	*Percentage of patients discontinuing treatment due to adverse events*	*14% (10%)*	*22*
*Treatment discontinuation due to adverse events* vs. *control*	*Difference in percentage of patients discontinuing treatment due to adverse events* vs. *control*	*8% (7%)*	*22*
*Median duration of treatment*	*Months (median) of duration of treatment*	*21.27 (24.54)*	*17*
*Other Indications (patients exposed)*	*Number of potential patients for all indications (exposed population as reported in EPAR)*	*920.95 (665.65)*	*22*
Comparative patient-perceived health and patient-reported outcomes			
*Quality of Life*	*Normalized score of quality-of-life scale*	*0.06 (0.22)*	*14*
*Impact on autonomy*	*Mentioned (yes/no) disruption of daily activities due to delivery of treatment*	*Yes: 41%* *No: 59%*	*22*
*Frequency of treatment (administration)*	*Dose administration by unit of time*	*Once a month: 4%* *Twice a month: 4%* *Once a week: 4%* *Twice a week: 0%* *>Twice a week: 9%* *Once a day: 48%* *Twice a day: 17%*	*22*
*Variable treatment guideline*	*Mentioned (yes/no) treatment guidelines changes*	*Yes: 13%* *No: 68%*	*22*
*Time of treatment*	*Mentioned (fixed/up to progression/variable) time of treatment*	*Fixed: 17%* *Up to progress: 50%* *Other: 36%*	*22*
*Easy to use, mode and set of administration*	*Mentioned (oral/injection/intrathecal) way of administration*	*Oral: 68%* *Injection: 27%* *Intrathecal: 4%*	*22*
*Combined chemotherapy*	*Mentioned (with/without) combination with chemotherapy*	*With: 18%* *Without: 81%*	*22*
Magnitude of therapeutic benefit (*)			
*Magnitude of clinical benefits MCBS*	*Scale of MCBS*	*3.14 (0.77)*	*22*
Type of benefit			
*Curative/Non-Curative*	*Mentioned (curative/non-curative) clinical benefit*	*Curative: 18%* *Non-Curative: 82%*	*22*
Comparative cost consequences – cost of intervention			
*NICE ICER > threshold*	*Mentioned (yes/no) NICE ICER > threshold before any patient access scheme was in place.*	*NA: 4%* *Yes: 73%* *No: 23%*	*21*
*NICE cancer fund*	*Mentioned (yes/no) inclusion as a NICE Cancer Fund’s Drug*	*Yes: 36%* *No: 64%*	*22*
*ICER (NICE value)*	*Δ monthly target therapy cost / Δ time to disease progression as per NICE information*	*52,363.9 (28,859.4)*	*18*
Comparative cost consequences – other medical costs			
*Cost treatment (procedures and tests-physician visits-hospitalizations…)*	*Yearly direct medical costs (€) excluding purchasing costs of the technology (i) concomitant medications, (ii) outpatient visits, diagnostic/laboratory tests, hospitalizations, and other monitoring costs (including management AEs), and (iii) terminal care.*	*NA: 50%* *0: 45%* *>0: 5%*	*11*
Comparative cost consequences – non-medical costs			
*Cost treatment*	*Yearly cost of (€) treatment (based on notified prices)*	*NA: 100%*	*0*
Quality of evidence (**)			
*JADAD scale*	*JADAD scale*	*2.50 (1.40)*	*22*
Expert consensus and clinical practice guidelines			
*Recommendation by experts*	*Mentioned (yes/no) recommendation included in consensus available at the time of pricing*	*Recommended: 24%* *Not recommended: 76%*	*17*
Contextual			
Mandate and scope of the healthcare system			
*Included in National/Sub-National Health Plan*	*Type of cancer mentioned (yes/no) in healthcare plans*	*Included: 82%* *Not included: 18%*	*22*
Population priorities and access			
*Preferences of the population as a need*	*Type of cancer mentioned (yes/no) in official positions or documents from NGO’s and Patient Advocacy Groups*	*Identified: 18%* *Not identified: 82%*	*22*
Common goal and specific interests			
*Stakeholders’ expression of interest and alignment*	*Type of cancer mentioned (yes/no) in societal sources (mass or digital media)*	*Identified: 23%* *Not identified: 77%*	*22*
Environmental impact			
*Impact of the intervention on environment – packaging, production*	*Relevant environmental impact mentioned (yes/no) in EPAR*	*Yes: 21%* *No: 79%*	*19*
System capacity and appropriate use of intervention			
*Healthcare services delivery change*	*Mentioned (yes/no) change in healthcare service delivery or inversion (*e.g.*, new biomarkers) to deliver care*	*Yes: 36%* *No: 64%*	*22*
Political, historical, or cultural context			
*Societal acceptability of the decisions*	*Type of cancer mentioned (yes/no) at legal level or included in political statements*	*Identified: 9%* *Not identified: 91%*	*22*

(*) Non-curative indications range from 1 (lowest) to 5 (highest) benefit. Curative indications range from A to C (A equalized to 5 and C to 1). (**) JADAD scores range from 0 (lowest) to 5 (highest) quality of trials.

Continuous variables for MCDA-EVIDEM dimension indicators were expressed as mean ± standard deviation, and categorical variables were expressed as percentages.

To evaluate the relation between oncology and hematology treatment prices and MCDA-EVIDEM indicators at the time of reimbursement decision, univariate analyses were performed. For correlation analyses, categories were normalized, summaries were calculated by dimension, and prices were categorized by terciles where required. To compare variables, the non-parametric Mann–Whitney test was used for continuous variables and the Fisher exact test was used for categorical variables. Spearman’s coefficients and 95% confidence intervals were calculated to assess correlations. The statistical definition was set at 5% two-tailed. The analysis was deemed exploratory, and, thus, no measures to account for multiplicity were applied.

## 3. Results

**Figure 1:**
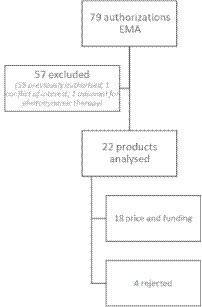
Product selection.

From January 2017 to December 2019, 24 oncological new chemical entities were granted a first indication marketing authorization in Europe. One product was excluded due to a conflict of interest in the team, and an adjuvant product for photodynamic therapy was deemed not suitable for the exercise (41) (Figure 1). Eventually, 22 products that aimed to treat 11 different tumors were analyzed. By October 2022, pricing and reimbursement had been granted for 18 products and denied for four products (Table 2). The most frequent indications were breast and lung cancer, and nine drugs had orphan designation (Table 2). Only two products had no therapeutic alternatives (in lung and agnostic indications) and roughly half of the products had targeted therapies as alternative options. Likewise, half of the treatments had an impact on patient autonomy (long intravenous administration, daycare admission), mostly in acute leukemia, lymphomas, melanoma, and neuroblastoma. Products for the treatment of melanoma, breast, neuroblastoma, and agnostic indications showed longer Progression Free Survival (PFS), observed and compared to control, over the median (14 months), and better Overall Survival (OS) vs. control was seen for products to treat leukemia and neuroblastoma. Most of the products were aimed at non-curative settings (19/22), with a moderate MCBS score (13/22 products under the score of 4) and low quality of evidence (17/22 products under a JADAD score of 3). Most did not require new healthcare service delivery routes (14/22) and were administered orally (15/22). Many had an Incremental Cost-Effectiveness Ratio (ICER) over the NICE threshold and were included in the NICE Cancer Drugs Fund (16/22), and most were related to cancers included in National or Regional Health Plans (18/22). More than half of the products (12/22) were explicitly recommended by expert consensus or included in clinical practice guidelines, while 4/22 products were explicitly not recommended (Table 1).

**Table 2:** Price and funding decisions by October 2022 for oncological products with first regulatory authorization* from January 2017 to December 2019.

Active principle	Indication	Date authorization	Date final P&R decision	Public funding	Time^#^ to final P&R decision (days)	Yearly treatment cost ~ (public listing price)
Inotuzumab ozogamicin	Acute lymphoblastic leukemia	21/07/2017	1/7/2019	yes	710	189,431.35 €
Dinutuximab beta	Neuroblastoma	06/09/2018	1/6/2022	yes	1,364	171,998.95 €
Mogamulizumab	Squamous cell carcinoma	05/06/2019	1/7/2021	yes	757	160,158.35 €
Polatuzumab vedotin	Acute myeloid leukemia	18/02/2020	1/9/2021	yes	561	139,200.05 €
Brigatinib	Lung cancer	28/11/2019	1/5/2021	yes	520	109,781.05 €
Durvalumab	Lung cancer	31/10/2018	1/1/2020	yes	427	98,550.00 €
Rucaparib	Breast cancer	10/05/2019	1/1/2020	yes	236	91,129.55 €
Midostaurin	Chronic myelogenous leukemia	30/10/2017	1/4/2019	yes	518	86,997.75 €
Encorafenib	Melanoma	04/10/2018	1/9/2019	yes	332	86,844.45 €
Binimetinib	Melanoma	19/10/2018	1/9/2019	yes	317	86,844.45 €
Niraparib	Ovarian cancer	08/03/2018	1/8/2019	yes	511	64,918.90 €
Lorlatinib	Lung cancer	20/06/2019	1/2/2021	yes	592	63,630.45 €
Neratinib	Breast cancer	07/01/2020	1/7/2022	no	906	61,320.00 €
Ribociclib	Breast cancer	04/09/2017	1/11/2017	yes	58	57,936.45 €
Tivozanib	Renal cancer	09/04/2018	1/3/2019	yes	326	47,650.75 €
Abemaciclib	Breast cancer	26/10/2018	1/5/2019	yes	187	46,668.90 €
Citarabine/ daunorubicin	Acute myeloid leukemia	19/12/2018	1/3/2022	yes	1.168	42,639.30 €
Gemtuzumab ozogamicin	Acute myeloid leukemia	25/05/2018	1/7/2019	yes	402	35,999.95 €
Dacomitinib	Lung cancer	23/05/2019	1/8/2020	yes	436	32,850.00 €
Talazoparib	Breast cancer	24/07/2019	1/8/2021	no	739	0.00 €
Gilteritinib	Acute myeloid leukemia	05/12/2019	1/6/2021	no	544	0.00 €
Larotrectinib	Agnostic indication	21/11/2019	1/4/2022	no	862	0.00 €

*Cemiplimab was excluded because of a conflict of interest; padeliporfin was excluded because the indication was an adjuvant for photodynamic therapy. ~ Cost calculated according to posology in the product information for the studied indication and annualized where required if fixed maximum length of treatment. Costs of 0.00 € reflect negative price and reimbursement decisions by October 2022. ^#^Time from the date of European Marketing Authorization until inclusion in the national reimbursement listing; since negative decisions and successive resubmissions may occur until reimbursement is granted, it does not reflect the length of pricing and reimbursement procedure.

The univariate analysis (Tables 3, 4) showed significantly higher listed prices when the standard of care was combined treatments, if long-lasting responders were reported, and for several characteristics of the treatment: higher prices for fixed-duration compared to treatment until progression and treatment with lower frequencies of administration, and lower prices for oral administration compared to other routes of administration. There were significant correlations between price and the ease of use of the drug, the impact of treatment on patient autonomy, and the existence of recommendations by experts. Regarding summaries by dimensions, the only association with price values was observed for the “expert consensus/clinical practice guidelines recommendations” dimension that contained a single item.

**Table 3:** Description of the mean (SD) listed yearly prices of oncology drugs according to the values of MCDA categorical items.

Variables and values	Mean	SD	Lower limit 95% CI	Upper limit 95% CI
Alternative treatment options				
With	78,800.06 €	52,983.23 €	55,308.61 €	131,783.30 €
Without	49,275.00 €	69,685.37 €	18,378.24 €	80,171.76 €
Type of standard of care				
Chemotherapy	68,353.67 €	66,410.51 €	38,908.90 €	97,798.44 €
Combined	143,946.84 €	43,194.75 €	124,795.36 €	163,098.32 €
Directed agents	59,858.69 €	29,376.64 €	46,833.81 €	72,883.56 €
None	49,275.00 €	69,685.37 €	18,378.24 €	80,171.76 €
Long responders				
Not mentioned	60,764.84 €	29,538.65 €	47,668.13 €	73,861.54 €
Yes	98,389.07 €	16,110.32 €	91,246.16 €	105,531.99 €
NA	89,928.88 €	75,408.73 €	56,494.52 €	123,363.23 €
Dosage adjustment due to AEs active				
No	86,236.67 €	80,786.88 €	50,417.77 €	122,055.56 €
Not Relevant	83,546.67 €	76,674.75 €	49,550.99 €	117,542.34 €
Yes	72,825.08 €	47,833.71 €	51,616.80 €	94,033.36 €
Impact of treatment on autonomy				
No	50,990.95 €	35,392.51 €	35,298.79 €	86,289.74 €
Yes	112,407.66 €	55,550.03 €	87,778.15 €	137,037.16 €
Interval of treatment administration				
Daily	55,771.42 €	35,111.77 €	40,203.73 €	71,339.10 €
Weekly or less frequent	104,747.50 €	71,259.64 €	73,152.75 €	136,342.25 €
Variable treatment guideline				
No	76,789.96 €	54,409.58 €	52,666.11 €	100,913.81 €
Yes	74,671.70 €	55,243.71 €	50,178.01 €	99,165.38 €
Duration of treatment				
Fixed schedule	110,623.33 €	56,319.09 €	85,652.85 €	135,593.82 €
Other	89,918.15 €	66,683.08 €	60,352.53 €	119,483.78 €
Up to progression	56,666.91 €	36,126.07 €	40,649.51 €	72,684.32 €
Easy to use/mode & set of administration				
Injection	108,091.67 €	58.881.72 €	81.984.97 €	134.198.36 €
Intrathecal	189,430.00 €	- €	- €	- €
Oral	55,771.42 €	35,111.77 €	40,203.73 €	71,339.10 €
Combined chemotherapy				
With	108,549.34 €	59,703.00 €	82,078.51 €	135,020.17 €
Without	68,908.55 €	50,841.26 €	46,366.80 €	91,450.30 €
ESMO – MCBS setting curative/non-curative				
Curative	89,079.34 €	59,071.05 €	62,888.70 €	115,269.98 €
Non-Curative	73,235.22 €	53,406.60 €	49,556.06 €	96,914.38 €
ICER (> NICE threshold)				
No	71,603.22 €	14,199.42 €	64,542.02 €	78,664.43 €
Yes	82,283.45 €	59,142.59 €	52,872.53 €	111,694.36 €
ICER (NICE cancer fund)				
No	81,547.28 €	62,812.10 €	53,697.95 €	109,396.60 €
Yes	66,611.17 €	32,409.99 €	52,241.39 €	80,980.96 €
Recommendation by experts				
NA	63,000.00 €	69,753.90 €	28,821.22 €	97,178.78 €
Not Recommended	35,209.46 €	26,193.69 €	22,374.79 €	48,044.14 €
Recommended	88,901.03 €	49,442.76 €	64,674.53 €	113,127.54 €
Included in national/sub-national health plan				
Included	73,752.18 €	49,723.26 €	51,706.12 €	95,798.24 €
Not Included	86,753.01 €	75,706.10 €	53,186.81 €	120,319.22 €
Preferences of the population as a need?				
Identified	110,990.61 €	45,170.96 €	90,962.93 €	201,953.55 €
Not identified	68,366.05 €	52,976.65 €	44,877.51 €	91,854.58 €
Stakeholders’ expression of interest & alignment				
Identified	98,322.90 €	48,297.69 €	76,908.91 €	119,736.90 €
Not identified	69,584.52 €	54,346.47 €	45,488.64 €	93,680.39 €
Impact of the intervention on environment – packaging, production				
NA	108,149.44 €	57,269.58 €	80,546.39 €	135,752.50 €
No	77,689.18 €	54,898.41 €	51,228.99 €	104,149.37 €
Yes	46,191.30 €	37,980.02 €	27,885.52 €	64,497.09 €
Healthcare services delivery change				
No	69,930.29 €	48,388.88 €	48,475.86 €	91,384.71 €
Yes	86,940.90 €	63,093.87 €	58,966.65 €	114,915.16 €
Societal acceptability of the decisions				
Identified	102,425.00 €	98,393.91 €	58,799.58 €	146,050.42 €
Not identified	73,485.06 €	50,562.17 €	51,067.05 €	95,903.07 €
All products				
Yearly price	76,115.97 €	53,353.38 €	52,460.40 €	99,771.53 €

SD, standard deviation; 95% CI, 95% Confidence interval; AEs, Adverse events; ESMO-MCBS, European Society of Medical Oncology – Magnitude of Clinical Benefit Score; ICER, Incremental Cost-effectiveness ratio; NICE, National Institute for Health and Care Excellence.

**Table 4:** Univariate analysis of the association between listed prices of oncology drugs and the dimensions of MCDA and subitems within each dimension.

Dimensions and individual items	N	Correlation estimate	Lower 95% confidence limit	Upper 95% confidence limit	Value of *p* for H0: Rho = 0
**1. Disease severity**	**22**	−0**0,29**	−0**0,63**	**0,15**	**0,18**
Speed tumor growth	20	−0.26	−0.61	0.18	0.23
% Metastasized	22	−0.23	−0.60	0.21	0.29
Expected survival 5-years	22	−0.37	−0.68	0.06	0.08
Overall Survival	20	0.09	−0.34	0.49	0.68
Physical function and general health (SF36 - EQ5D - EORTC QLQ-C30)	12	−0.11	−0.50	0.33	0.63
**2. Size of affected population**	**22**	**0,17**	**−0,27**	**0,55**	**0,44**
Prevalence	22	0.23	−0.21	0.59	0.30
Incidence	22	0.16	−0.28	0.54	0.47
**3. Unmet needs**	**22**	**0,05**	**−0,38**	**0,46**	**0,81**
Treatment options	22	−0.07	−0.48	0.36	0.74
Type of standard of care	22	0.05	−0.38	0.46	0.81
**4. Comparative effectiveness**	**22**	**0,15**	**−0,29**	**0,54**	**0,50**
Progression-Free Survival observed	22	−0.14	−0.53	0.30	0.53
Progression-Free Survival difference compared to control	18	0.14	−0.30	0.53	0.52
Objective Response Rate (RECIST/MRD) observed	19	−0.35	−0.67	0.09	0.10
Objective Response Rate (RECIST/MRD) difference compared to control	14	0.20	−0.24	0.57	0.37
Complete response (RECIST/MRD) observed	20	−0.01	−0.43	0.41	0.96
Complete response (RECIST/MRD) difference compared to control	15	0.38	−0.04	0.69	0.07
Partial response (RECIST /MRD) observed	18	−0.15	−0.54	0.29	0.49
Partial response (RECIST /MRD) difference compared to control	13	0.27	−0.17	0.62	0.22
Long responders (Yes/no)	11	0.17	−0.27	0.55	0.44
Overall Survival observed	15	0.21	−0.23	0.58	0.33
Overall Survival difference compared to control	12	0.29	−0.15	0.63	0.18
**5. Comparative safety/tolerability**	**22**	**−0,13**	**−0,53**	**0,30**	**0,55**
Any Adverse Events observed	22	−0.18	−0.56	0.26	0.42
Any Adverse Events difference compared to control	16	0.04	−0.39	0.45	0.87
Non-Fatal Serious Adverse Events (>3) observed	22	0.15	−0.29	0.54	0.50
Non-Fatal Serious Adverse Events (>3) difference compared to control	16	−0.02	−0.44	0.40	0.91
Fatal Adverse Events (Grade 5 AEs) observed	21	−0.06	−0.47	0.37	0.78
Fatal Adverse Events (Grade 5 AEs) difference compared to control	16	0.08	−0.35	0.48	0.72
Dosage adjustment due to adverse effects	22	0.06	−0.37	0.47	0.78
Treatment discontinuation (due to AEs) active	22	−0.25	−0.61	0.19	0.25
Treatment discontinuation (due to AEs) difference compared to control	17	−0.07	−0.48	0.35	0.74
Median duration of treatment	22	−0.49	−0.75	−0.09	**0.01**
Extent of exposure: Other indications, number of indications	22	−0.22	−0.58	0.22	0.31
**6. Comparative patient-perceived health/PRO**	**22**	**−0,14**	**−0,53**	**0,30**	**0,54**
HRQoL	14	0.37	−0.06	0.68	0.08
Impact on Autonomy	22	−0.45	−0.73	−0.04	**0.03**
Frequency of treatment (administered how often)	22	0.40	−0.03	0.70	0.06
Variable treatment schedule	22	0.02	−0.40	0.44	0.92
Time of treatment	22	0.41	−0.01	0.71	0.05
Easy to Use/Mode & Set of Administration	22	−0.48	−0.75	−0.08	**0.02**
Combined chemotherapy	22	−0.27	−0.62	0.17	0.21
**7.a. Magnitude of preventive benefit**	**18**	**0.16**	**−0.28**	**0.55**	**0.47**
Magnitude of preventive benefit	18	0.16	−0.28	0.55	0.47
**7.b. Magnitude of therapeutic benefit**	**22**	**0.13**	**−0.31**	**0.52**	**0.57**
Magnitude of therapeutic benefit	22	0.13	−0.31	0.52	0.57
**8. Comparative cost consequences – cost of intervention**	**22**	**−0,03**	**−0,45**	**0,39**	**0,87**
Incremental Cost-effectiveness ratio (ICER) over NICE threshold (yes/no)	21	−0.09	−0.49	0.34	0.69
ICER: NICE assigns cancer fund (yes/no)	22	−0.01	−0.43	0.41	0.95
ICER: NICE value (€ or pounds – with 95% CI)	18	−0.08	−0.49	0.35	0.70
**11. Quality of evidence:**	**22**	**−0,02**	**−0,44**	**0,40**	**0,91**
JADAD/ESMO assessment of quality (from 1 to 5, where 5 is the maximum)	22	−0.02	−0.44	0.40	0.91
**12. Expert consensus/clinical practice guidelines**	**17**	**0,56**	**0,17**	**0,79**	**0,00**
Availability of guidance for use and recommendation in guidance/by experts	17	0.56	0.17	0.79	*0.00*
**13. Contextual criteria**	**22**	**0,03**	**−0,40**	**0,44**	**0,90**
Mandate and scope of the healthcare system	22	−0.05	−0.46	0.38	0.81
Population priorities and access	22	0.35	−0.09	0.67	0.11
Common goal and specific interests	22	0.26	−0.18	0.61	0.24
Environmental impact	19	0.01	−0.41	0.43	0.97
System capacity and appropriate use of intervention	22	−0.17	−0.55	0.26	0.43
Political/historical/cultural context	22	0.05	−0.38	0.46	0.83

Dimension 7 was analyzed separately for preventive and therapeutic benefits since these used different scoring. Dimensions 9 to 12 had a single item each, so the estimate for the dimension is the same as that of the item. Due to lack of data, dimensions number 9 “comparative cost consequences – other medical costs” and the corresponding item “Cost treatment (procedures and tests-physician visits-hospitalizations) / Year” and number 10 “comparative cost consequences –non-medical costs” and the corresponding item “Cost/ Year” were not analyzed for correlation.

## 4. Discussion

Our findings suggest that the initial price of oncology and hematology products tends to be influenced (higher prices) only by a few variables: the type of standard of care, the reporting of long-lasting responders, the convenience of use of drugs, the impact on patient autonomy, a limited duration of treatment, and contextual indicators such as the existence of previous clinical consensus. None of the individual items for comparative efficacy, safety, or quality of life reached significance for price correlation. Attempts to summarize values by dimensions compared to descriptions of individual items did not improve the explanation of price differences. However, the lack of standardized metrics and harmonized interpretation of contextual indicators limits the interpretation of the results.

The main limitation to moving forward with more transparent and standardized drug pricing processes is the lack of shared convention about the definition of “price” as an expression of “value” (1). For example, concepts such as quality-adjusted life years (to standardize health gains) do not capture the social perception of health benefits when the life expectancy of diseases differs (42). Additionally, price-setting processes are conditioned by available and previous therapeutic alternatives, influencing prices of pharmaceutical innovation based on historical inertia and baseline costs of the disease for the system (43). Additionally, dose, posology, and treatment duration add complexity to the direct comparison of value-based prices of new drugs.

There is a diversity of standardized clinical outcomes (overall survival, progression-free survival, quality of life, and safety) that medical societies and European healthcare authorities (38) are using to guide or define reimbursement conditions of oncology drugs (44). Other reports (17, 19–21, 23, 45) suggest that perceived additional therapeutic benefits based on weak variables (such as response rates) or perception of severity (when this is measured) may be driving oncology drug prices. In our data, these clinical variables and “hard” variables such as overall survival were not good pricing predictors. However, we observed higher prices for products reporting references to long-lasting responders. Furthermore, our research also shows that other intermediate indicators such as PFS, generally accepted as indicators of the capacity of a drug to cure or alter the natural history of the disease (46), were not strong predictors of prices either. The lack of consistent evidence based on long-term efficacy data or relative efficacy data of new drugs vs. frequently used drugs at the time of price negotiations does not seem to have any penalty on the price and reimbursement decisions in Spain. The study also suggests the influence of contextual indicators, such as the existence of expert consensus and the impact of the route of administration to patients, on setting prices.

Several limitations of the study should be considered. Firstly, only a few new oncology drugs authorized for a first indication were analyzed. The influence that multiple indications may have in price negotiations requires further analysis. Secondly, the value assessment was made by evaluators working in the context of payers of healthcare services, so that may not fully reflect the perspectives of pricing and reimbursement decision-making. Third, we did not calculate summary indicators or overall scores for MCDA-EVIDEM, as suggested by others (43), since the exercise aimed to verify whether a more transparent reporting of the criteria used for decisions may help all stakeholders to predict the key determinants of value and to support the expectations of manufacturers, the information given to the lay public, and the consistency of the decision-making by authorities. Finally, we did not run a systematic search of the literature using a diverse range of databases to identify all potential studies analyzing the relationship between prices and the MCDA-EVIDEM framework, and there is a scarcity of references available on methods and definitions for data extraction and analysis; therefore, we cannot exclude that our work may be influenced by publication biases.

Our work may provide a basis for some proposals in the context of upcoming regulations and changes in Health Technology Assessments. The new European regulation (47) states that inclusive joint clinical assessments able to respond to all Member States’ requirements must be produced at the EU level, ideally through consensus, and become part of multi-step national procedures. This new regulation enhances, in this way, the relevance of multiple domains (clinical, social, or economic) of assessment in the process of decision-making by national price and reimbursement organisms, EVIDEM being a solid starting point. From this perspective, further research is needed to standardize measures and determine the socially acceptable weights among EVIDEM dimensions, as well as its translation into economic values by dimension. So far, very limited experiences (48) have been tested with this broader approach aimed at more transparent and fair pricing but there is still a lack of solutions to tackle additional limitations, such as a potential disincentive effect on R&D efficiency discouraging future disruptive innovation.

## 5. Conclusion

Our exercise shows that, regardless of the paucity of explicative criteria on the decisions, the use of a standardized multidimensional framework allowed us to identify that the listed prices of new cancer products with a single first reimbursed indication in Spain are related to the type of standard of care, references to long-lasting responses, the convenience of use of the drug, and its impact on patient’s autonomy, as well as contextual indicators such as the existence of previous clinical consensus. While individual items are relatively explanatory, grouping by the synthetic MCDA-EVIDEM dimensions does not improve explicative value or information.

Based on our results and the lack of detailed information on how Spanish healthcare authorities define price and reimbursement conditions of new onco-hematologic drugs, we propose that the implementation of MCDA-EVIDEM methodologies may help to capture and report additional factors generally not included in consolidated assessment frameworks, such as the European Network for Health Technology Assessment (EunetHTA) core model. It may be opportune to consider this in the upcoming revision of the Spanish regulation for health technology assessments and pricing and reimbursement procedures (49).

## Data availability statement

The datasets presented in this study can be found in online repositories. The names of the repository/repositories and accession number(s) can be found in the article/Supplementary material.

## Author contributions

DE: Conceptualization, Data curation, Formal analysis, Investigation, Methodology, Project administration, Resources, Supervision, Validation, Visualization, Writing – original draft, Writing – review & editing. FT: Conceptualization, Data curation, Investigation, Methodology, Validation, Writing – review & editing. RV: Conceptualization, Data curation, Formal analysis, Investigation, Methodology, Validation, Writing – review & editing. GP: Conceptualization, Data curation, Formal analysis, Investigation, Methodology, Validation, Writing – review & editing. MO: Conceptualization, Data curation, Formal analysis, Investigation, Methodology, Validation, Writing – review & editing. DG: Conceptualization, Data curation, Formal analysis, Investigation, Methodology, Writing – review & editing. DV: Conceptualization, Data curation, Investigation, Methodology, Writing – review & editing. TP: Conceptualization, Data curation, Formal analysis, Investigation, Methodology. JT: Conceptualization, Supervision, Validation, Writing – review & editing. CP: Conceptualization, Data curation, Formal analysis, Investigation, Methodology, Supervision, Validation, Writing – review & editing.

## Abbreviations

AEMPS: Spanish Agency of Medicines and Medical Devices
CIPM: Inter-ministerial Committee on Pricing of Medicines and Healthcare Products
DGCCSF: General Directorate for Common Portfolio of the NHS and Pharmacy Services
EPAR: European Public Assessment Report
ESMO-MCBS: European Society of Medical Oncology-Magnitude of Clinical Benefit Scale
EUnetHTA: European Network for Health Technology Assessment
EVIDEM: Evidence and Value Impact on Decision-Making
HTAb: Health Technology Assessment Bodies
ICER: Incremental Cost-Effectiveness Ratio
MCDA: Multi-Criteria Decision Analysis
MoH: Ministry of Health
NHS: National Health Service
NICE: National Institute for Health and Care Excellence
OS: Overall Survival
PFS: Progression-Free Survival
QALYS: Quality-Adjusted Life-Years
TPR: Therapeutic Positioning Report
