# Supplementary material for: Reporting reimbursement price decisions for onco-hematology drugs in Spain
Source: Front Public Health. 2023 Oct 24;11:1265323. doi: 10.3389/fpubh.2023.1265323 (PMC10627880; doi:10.3389/fpubh.2023.1265323)
Supplement: Supplementary file 2 [file Table_2.DOCX]

*Supplementary Material*

Table 1 – Source of MCDA-EVIDEM indicators from literature review

| ***Source*** | ***Dimension*** | ***Indicator*** | ***Definition / Assumptions*** |
| --- | --- | --- | --- |
| *Angelis A. et al. Social Science & Medicine. 2020; 246;112595.*  *Angelis A. et al. Multiple criteria decision analysis in the context of health technology assessment: a simulation exercise on metastatic colorectal cancer with multiple stakeholders in the English setting. BMC Medical Informatics and Decision Making; 17:149. DOI 10.1186/s12911-017-0524-3.2017.* | *Therapeutic Benefit* | *Overall survival x Health related quality of life* | *The median time from treatment randomization to death adjusted for the mean health related quality of life using the EQ-5D utility score* |
|  |  | *Radiographic tumor progression* | *The median survival time on which patients have not experienced disease progression (using RECIST criteria)* |
|  |  | *PSA response* | *The proportion of patients having a ≥50% reduction in PSA* |
|  | *Safety Profile* | *Treatment discontinuation* | *The proportion of patients discontinuing treatment due to adverse events* |
|  |  | *Contra-indications* | *The existence of any type of contra-indication accompanying the treatment* |
|  | *Patient convenience* | *Delivery posology* | *The combination of the delivery system (RoA and dosage form) with the posology (frequency of dosing and duration of administration) of the treatment* |
|  |  | *Special instructions* | *The existence of any special instructions accompanying the administration of the treatment* |
|  | *Socioeconomic Impact* | *Medical costs impact* | *The impact of the technology on direct medical costs excluding the purchasing costs of the technology* |
| *Hsu JC. Et al. (2019) Comprehensive value assessment of drugs using a multi-criteria decision analysis: An example of targeted therapies for metastatic colorectal cancer treatment. PLoS ONE 14(12): e0225938.* [*https://doi.org/10.1371/journal.pone.0225938*](https://doi.org/10.1371/journal.pone.0225938) | *Efficacy* | *Overall survival period* | *“head-to-head” comparison or not / for first-line clinical situation use or not / hazard ratios are significant or not* |
|  | *Comparative safety* | *Overall incidence of adverse events* | *Percentage (%)* |
|  |  | *Incidence of adverse events (over Grade 3)* | *Percentage (%)* |
|  |  | *Dosage adjustment for special groups* | *Need adjustment (Yes/No)* |
|  |  | *Drug-drug interaction* | *Yes/No* |
|  | *Convenience and quality of life* | *Formulation* | *Oral / Injection* |
|  |  | *Frequency of use* | *-* |
|  |  | *Combined chemotherapy prescription* | *With/without impacts* |
|  |  | *Treatment duration* | *-* |
|  |  | *Quality of life* | *With/without impacts* |
|  | *Economic impact* | *ICER* | *Δ monthly target therapy cost / Δ time to disease progression.*  *Compare medicines with similar status (ex. the same line of treatment); Consider the cost for side effect treatment* |
|  |  | *Number of patients who would use this medicine* | *Consider gene-type variance ratios and clinical treatment options* |
|  |  | *Indications* | *With/without impacts* |
|  |  | *Overall target therapy expenditures* | *number of patients * treatment cost * duration; Count total costs* |
|  | *Societal impact* | *Mechanism* | *With/without innovation* |
|  |  | *Irreplaceability* | *With/without alternative drugs* |
|  |  | *Recommendation for the coverage by Health Technology Assessment (HTA) Reports* | *For first- and second-line treatments* |
|  |  | *Countries offering insurance coverage for the drugs* | *Coverage or not* |
| *Trotta F, Mayer F, Barone-Adesi F, et al. Anticancer drug prices and clinical outcomes: a cross-sectional study in Italy. BMJ Open 2019;9:e033728. doi:10.1136/bmjopen-2019-033728* | *Clinical benefit* | *Median overall survival (OS)* | *-* |
|  |  | *Median progression-free survival (PFS)* | *-* |
|  |  | *Objective response rate (ORR)* | *-* |
| *Wagner M. et al. (2018) Applying Reflective Multicriteria Decision Analysis (MCDA) to Patient–Clinician Shared Decision-Making on the Management of Gastroenteropancreatic Neuroendocrine Tumors (GEP-NET) in the Spanish Context. Adv Ther; 35:1215–1231.* [*https://doi.org/10.1007/s12325-018-0745-6*](https://doi.org/10.1007/s12325-018-0745-6) | *Efficacy / Effectiveness* | *Progression-free survival (PFS)* | Reference to [*https://www.evidem.org/*](https://www.evidem.org/)*.( Accessed by authors on 17 Sep 2015).* |
|  |  | *Disease symptoms* |  |
|  |  | *Tumor regression rate* |  |
|  |  | *Overall survival (OS)* |  |
|  | *Patient convenience / PRO* | *HRQoL* |  |
|  |  | *Impact on autonomy* |  |
|  |  | *Impact on dignity* |  |
|  |  | *Convenience/ease/setting* |  |
|  | *Safety / Tolerability* | *Non-fatal non-serious AEs* |  |
|  |  | *Non-fatal serious AEs* |  |
|  |  | *Fatal AEs* |  |
|  | *Type of benefit* | *Therapeutic benefit* |  |
|  |  | *Preventive benefit* |  |
|  | *Need for intervention* | *Disease severity* |  |
|  |  | *Unmet needs* |  |
|  |  | *Size of affected population* |  |
|  | *Economic impact* | *Cost of intervention* |  |
|  |  | *Other medical costs* |  |
|  |  | *Non-medical costs* |  |
|  | *Knowledge* | *Quality of evidence* |  |
|  |  | *Expert consensus / CPGs* |  |
|  | *Feasibility* | *System capacity / Appropriate use* |  |
